# 3D inversion recovery ultrashort echo time MRI can detect demyelination in cuprizone-treated mice

**DOI:** 10.3389/fnimg.2024.1356713

**Published:** 2024-05-09

**Authors:** Adam C. Searleman, Yajun Ma, Srihari Sampath, Srinath Sampath, Robert Bussell, Eric Y. Chang, Lisa Deaton, Andrew M. Schumacher, Jiang Du

**Affiliations:** ^1^Department of Radiology, University of California, San Diego, San Diego, CA, United States; ^2^Radiology Service, Veterans Affairs San Diego Healthcare System, San Diego, CA, United States; ^3^Novartis Institutes for BioMedical Research, San Diego, CA, United States; ^4^Department of Bioengineering, University of California, San Diego, San Diego, CA, United States

**Keywords:** MRI, IR-UTE, myelin imaging, mouse model, cuprizone

## Abstract

**Purpose:**

To test the ability of inversion-recovery ultrashort echo time (IR-UTE) MRI to directly detect demyelination in mice using a standard cuprizone mouse model.

**Methods:**

Non-aqueous myelin protons have ultrashort T_2_s and are “invisible” with conventional MRI sequences but can be detected with UTE sequences. The IR-UTE sequence uses an adiabatic inversion-recovery preparation to suppress the long T_2_ water signal so that the remaining signal is from the ultrashort T_2_ myelin component. In this study, eight 8-week-old C57BL/6 mice were fed cuprizone (*n* = 4) or control chow (*n* = 4) for 5 weeks and then imaged by 3D IR-UTE MRI. The differences in IR-UTE signal were compared in the major white matter tracts in the brain and correlated with the Luxol Fast Blue histochemical marker of myelin.

**Results:**

IR-UTE signal decreased in cuprizone-treated mice in white matter known to be sensitive to demyelination in this model, such as the corpus callosum, but not in white matter known to be resistant to demyelination, such as the internal capsule. These findings correlated with histochemical staining of myelin content.

**Conclusions:**

3D IR-UTE MRI was sensitive to cuprizone-induced demyelination in the mouse brain, and is a promising noninvasive method for measuring brain myelin content.

## 1 Introduction

In the central nervous system, myelin is a component of oligodendrocytes defined by its ultrastructure of multiple lamellae of protein-rich lipid bilayers; myelin insulates the axons of certain nerves to facilitate saltatory conduction and provides trophic support (Morell and Quarles, 1999a). The extent of myelination modulates the function and health of axons and is dynamically regulated throughout the lifespan and in response to a variety of neurologic conditions (Young et al., 2013; Duncan and Radcliff, 2016).

The development of robust and specific biomarkers of myelination would facilitate further advancements in the diagnosis, monitoring, and treatment of demyelinating diseases. For instance, there has been recent interest in the development of promoters of remyelination as complementary to the current immunosuppressive treatment of multiple sclerosis (Magalon et al., 2012; Deshmukh et al., 2013; Plemel et al., 2017; Lubetzki et al., 2020), an autoimmune demyelinating disease that most commonly affects young adults and ultimately results in progressive functional impairment, cognitive deficits, and early mortality (Lucchinetti et al., 2005; Popescu et al., 2013). An imaging-based biomarker of myelin would be crucial for the development of these potential remyelinating agents at both the pre-clinical and clinical stages.

Myelin imaging is challenging because the T_2_ of myelin protons (T_2_ from several μs to a few 100's of μs) is much shorter than the minimal echo times (TEs; no less than several ms) of conventional sequences (Horch et al., 2011; Wilhelm et al., 2012; Du et al., 2014a; Sheth et al., 2016). In addition, the longer T_2_ water protons comprise over 90% of the MRI signal in the brain (Fan et al., 2018). We have previously shown that adiabatic inversion recovery prepared ultrashort echo time (IR-UTE) sequences with nominal TEs as short as 8 μs are able to robustly suppress long-T_2_ water signals and generate a high contrast myelin image on a clinical 3T scanner (Du et al., 2014a,b). This short T_2_ signal persists after removing most of the myelin-associated water fraction by D_2_O exchange (Fan et al., 2017, 2018; Seifert et al., 2017), suggesting that the IR-UTE sequence is detecting signal from semisolid myelin protons. Myelin is comprised of ~40–45% lipids/cholesterol, 10–15% protein, and 40% water (Morell and Quarles, 1999b). The majority of the myelin protons detected by UTE sequences are thought to originate from the long-chain methylenes of the bilayers, with additional contributions from cholesterol, choline, and proteins (Horch et al., 2011; Wilhelm et al., 2012). The IR-UTE sequence has also been shown to detect demyelinated MS lesions as confirmed by autopsy (Sheth et al., 2016, 2017).

This study was designed to test the capability of the IR-UTE sequence in detection of demyelination using an animal model. In adult C57BL/6 mice, the copper chelator cuprizone induces demyelination with well-characterized temporal and regional dynamics, notably resulting in pronounced demyelination of the caudal corpus callosum (CC) after 5–6 weeks of exposure (Hiremath et al., 1998; Mason et al., 2001; Matsushima and Morell, 2001; Taylor et al., 2010). Additional acute changes are likely reactive to demyelination including mild edema, microgliosis, astrocytosis, and axonal injury; however there is minimal inflammation compared to models such as experimental autoimmune encephalitis (Matsushima and Morell, 2001; Gudi et al., 2009). Thus, the C57BL/6 cuprizone model is well-suited for determining the sensitivity of IR-UTE for acute demyelination.

In this study, eight 8-week-old C57BL/6 mice were fed cuprizone (*n* = 4) or control chow (*n* = 4) for 5 weeks and then imaged by 3D IR-UTE MRI and compared with conventional diffusion tensor imaging (DTI). The differences in IR-UTE signal were compared in the major white matter tracts in the brain and correlated with the Luxol Fast Blue (LFB) histochemical marker of myelin.

## 2 Materials and methods

### 2.1 Sample preparation

All animal studies conformed to institutional IACUC-approved protocols. Ten 8-week-old female C57BL/6 mice were included in this study. Five mice were given 0.2% cuprizone chow (Sigma Aldrich, St Louis, MO; Harlan Laboratories, Inc., Madison, Wisconsin) *ad lib* for 5 weeks, and five controls were given chow lacking cuprizone for 5 weeks prior to being sacrificed for imaging and analysis. This cuprizone dose and duration were chosen to induce maximal regional demyelination in the caudal corpus callosum in this mouse strain (Horch et al., 2011; Wilhelm et al., 2012; Du et al., 2014a; Sheth et al., 2016). One mouse from each group was used for image optimization and excluded from further analysis due to extended imaging times, which may have altered the biological characteristics of the myelin and surrounding tissues. The mice were decapitated and their heads skinned and then flash frozen in liquid nitrogen until analysis to prevent temporal effects related to scan order. Each head was warmed in a room temperature water bath for 2 h immediately prior to MRI to allow for a consistent brain temperature between specimens given the effect of temperature on T_1_ and thus nulling time.

### 2.2 MRI

Brain imaging was performed on a Bruker 7T BioSpec (Billerica, MA) scanner using a mouse brain surface coil for signal reception. The specimens were placed in 15 mL conical tubes on a cardboard insert to facilitate consistent positioning in the center of the coil. No solution was added to the tubes. A conventional T_2_-weighted fast spin echo (T_2_-FSE) sequence, with repetition time (TR) = 2,760 ms, TE = 40 ms, and echo train length (ETL) = 8, was used for anatomic imaging. A conventional two-dimensional adiabatic inversion recovery prepared FSE (2D IR-FSE) sequence, with TR = 8,000 ms, TE = 18.6 ms, and inversion times (TIs) = 60, 150, 300, 450, 600, 750, 900, 1,200, 1,500, and 2,000 ms, was used to measure T_1_ of the long T_2_ white matter components using a single coronal image of the ventral hippocampal commissure. A 3D IR-UTE sequence was used to image myelin, using the following parameters: TR = 1,000 ms, TI = 382.5 ms, TE = 20 μs, FOV = 2.0 × 2.0 × 2.0 cm^3^, matrix = 110 × 110 × 110, flip angle = 15°, number of excitations (NEX) = 4. To speed up data acquisition, 25 spokes centered on TI were acquired per IR preparation, with 5 ms from the start of one spoke to the next, leading to a total scan time of 100 min. The same 3D IR-UTE sequence was repeated with TE = 2.0 ms and NEX = 1. Similar imaging parameters were used for the T_2_-FSE and IR-FSE sequences. Echo planar imaging based DTI MRI was performed using 30 non-colinear gradient directions with gradient b-values = 0, 750, 1,500, and 2,000 s/mm^2^, TR = 4,000 ms, TE = 23 ms, and ETL = 13, for a total scan time of 25 min.

### 2.3 Histology

Immediately after imaging, mouse brains were removed intact, fixed in zinc formalin, and then embedded in paraffin using standard protocols as previously described (Beckmann et al., 2018). One of the control mouse brains was damaged during sample processing and excluded from further histological analysis. Five micron sections were stained with LFB using standard protocols as previously described (Beckmann et al., 2018). Myelin content was semi-quantitatively measured in the genu of the corpus callosum based on staining density as follows: the corpus callosum in each section was visually identified and outlined as the region of interest (ROI) for analysis using the NDP View software for NanoZoomer scanners (Hamamatsu, Photonics, Shizuoka, Japan). The myelin density was then calculated as the LFB optical density within this ROI. LFB densities from nine corpus callosum sections per animal were averaged, and this was compared across 3–4 animals per group.

### 2.4 Region of interest selection

The 3D IR-UTE images of a control mouse were mapped to anatomical structures using the Allen Mouse Brain Atlas as a reference (Allen Institute for Brain Science, 2004). Regions of interest (ROIs) were generated in two ways. First, ROIs were drawn manually for the genu and splenium of the CC, ventral hippocampal commissure (VHC), and internal capsule by an investigator blinded to group assignment using standardized criteria for ROI selection to ensure consistency. Secondly, ROIs were generated in a semi-automated fashion from a common IR-UTE template. To remove the influence of signal from the calvarium and other extracranial structures on the registration method, brain extraction was performed using custom Matlab scripts (The Mathworks Inc., Natick, MA). The IR-UTE template was generated using Advanced Normalization Tools (Avants et al., 2008) using symmetric diffeomorphic image registration with a cross-correlation metric from IR-UTE images of an untreated mouse. Each specimen was individually registered to this common template. The ROI analysis was done by manually drawing the ROI on the template and using the inverse transformation to map to the corresponding ROI in the original image space. Both ROI analyses were comparable; however, the backpropagation of the ROIs was not as reliable and therefore the first analysis is presented as it was considered to be more accurate. The template-registered IR-UTE images were also analyzed by averaging the registered IR-UTE images in the control mice and the cuprizone-treated mice to obtain averaged IR-UTE signal for each voxel. Subtraction of the averaged control IR-UTE map from the averaged cuprizone-treated IR-UTE map will demonstrate differences in signal for each voxel between these two groups, which was then analyzed for regional patterns of change.

### 2.5 Data analysis

The IR-UTE absolute signal was obtained from magnitude images. A coil sensitivity map was generated using the magnitude images from the same IR-UTE sequence on a degassed phantom of 20% H_2_O and 80% D_2_O with 35.5 mM MnCl_2_ (for ${\text{T}}_{2}^{*}$ of 355 ms) in a 15 mL conical tube. The map was then smoothened with a Gaussian filter with size = 4 and σ = 2 and scaled to a maximum value of 1. The IR-UTE signal from each specimen was normalized to this coil sensitivity map by simple division. The fractional anisotropy (FA), axial diffusivity (AD), and radial diffusivity (RD) were generated using the *dtifit* function in FDT (Jbabdi et al., 2012), and compared to the IR-UTE signal using the Pearson correlation coefficient. T_1_ values of white matter were calculated using custom code in Matlab in the ventral hippocampal commissure using the normalized maximum likelihood estimate (assuming a Rician distribution) and non-linear least squares fitting. Statistical analysis was performed using the R statistical programming language (v3.4.1) using the Wilcoxon rank-sum test for each ROI. A *p*-value of < 0.05 was considered statistically significant.

## 3 Results

**Figure 1:**
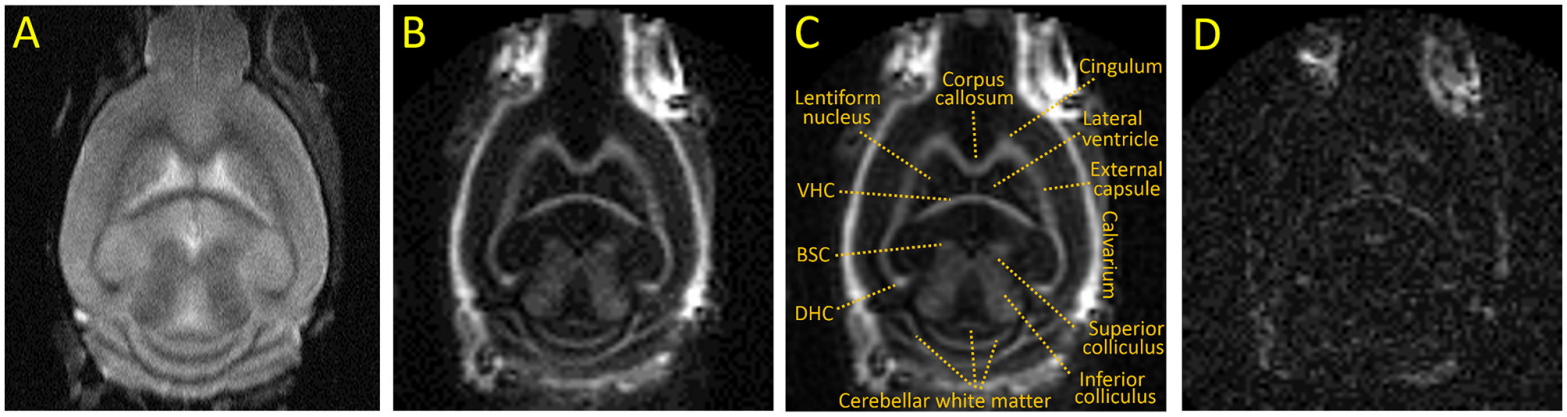
Representative coronal images of an untreated adult C57BL/6 mouse. **(A)** T2 weighted FSE, **(B, C)** IR-UTE at TE = 0.020 ms with and without annotations, **(D)** IR-UTE at TE = 2 ms [displayed with a 10X narrower window than **(B)** to show detail]. BSC, Brachium of the superior colliculus; DHC and VHC, dorsal and ventral hippocampal commissure, respectively.

Representative images of the optimized IR-UTE sequence of a control mouse brain are shown in Figure 1. The IR-UTE image in Figures 1B, C demonstrates high signal intensity in the major white matter tracts, including the corpus callosum, internal capsule, dorsal and ventral hippocampal commissures, and deep cerebellar white matter; intermediate signal in mixed white and gray matter structures such as the basal ganglia and both superior and inferior colliculi; and minimal signal from cortical gray matter and cerebral spinal fluid (CSF). The high signal in the calvarium seen only on IR-UTE and not FSE images reflects ultrashort T_2_ signal from cortical bone, and there is additional signal from retrobulbar fat. The later echo time of TE = 2 ms in Figure 1D has dramatically reduced signal intensities, notably with the majority of the white matter tracts now demonstrating less signal than residual CSF, indicating that the majority of the white matter IR-UTE signal has ultrashort T_2_. The retrobulbar fat, bone marrow fat, and minimal CSF signal are more apparent in the TE = 2 ms image due to longer T_2_s.

**Figure 2:**
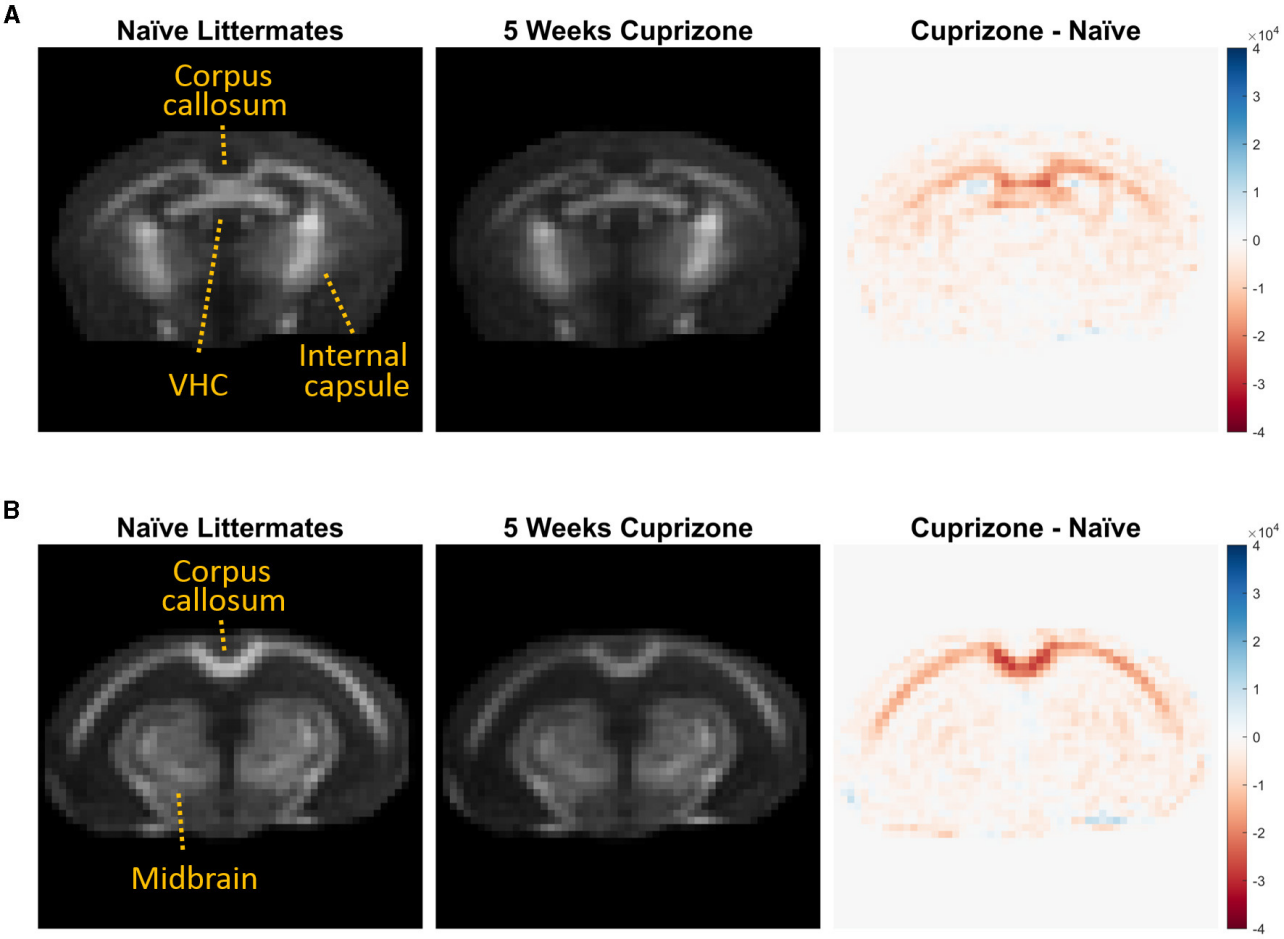
Averaged IR-UTE signal from untreated and cuprizone treated mice. IR-UTE images were registered to a common template after brain extraction and then averaged. These averaged axial images are displayed at the level of the **(A)** ventral hippocampal commissure and **(B)** splenium corpus callosum. On the right, subtraction images are shown such that areas of decreased signal in the cuprizone-treated mice are red, and areas of no change are white. Note that the largest decrease in signal was in the splenium of the corpus callosum **(B)**, and the lack of signal differences in the internal capsule **(A)** and midbrain structures **(B)** that are resistant to cuprizone.

**Figure 3:**
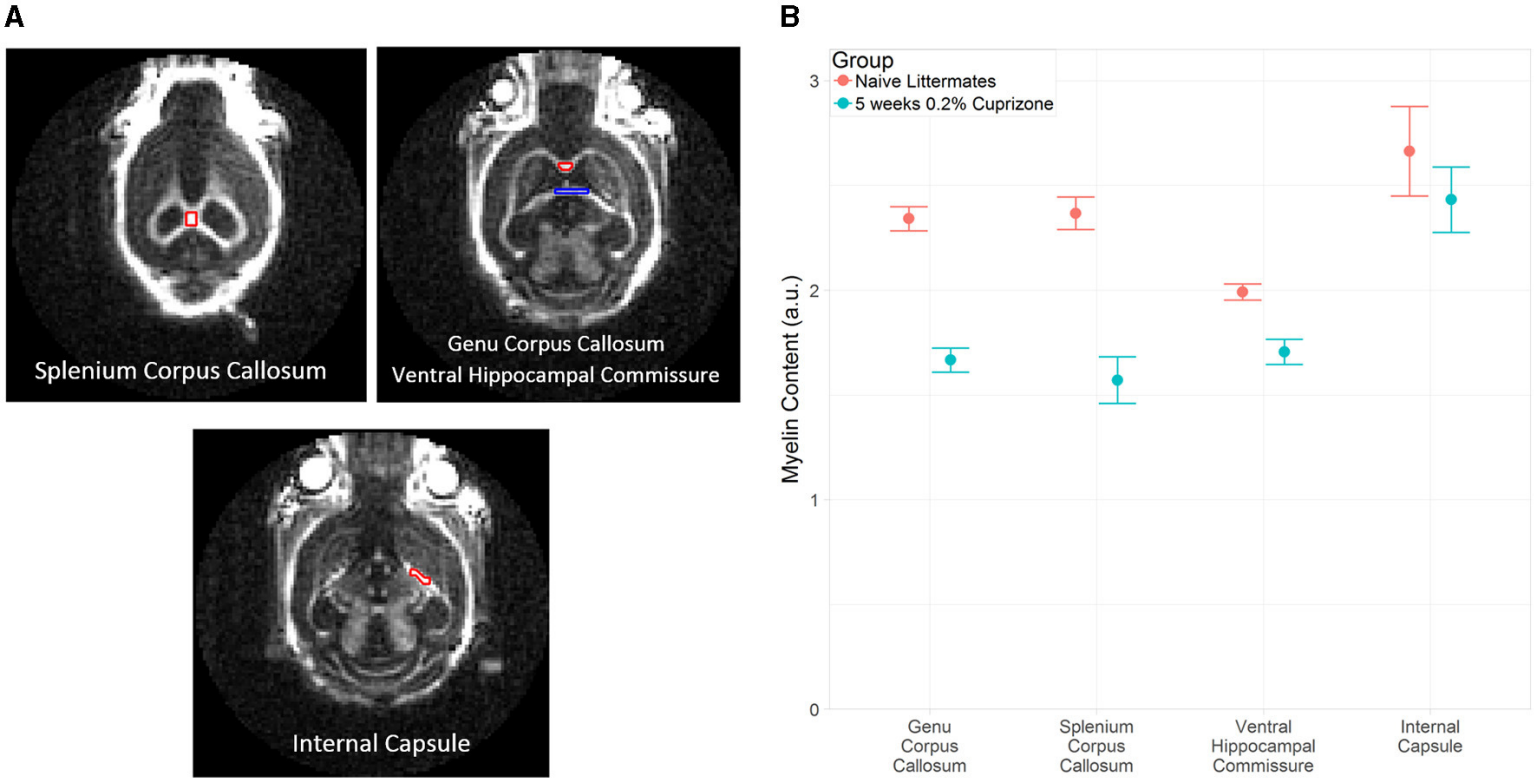
**(A)** Example of ROI placement in a representative control mouse on coronal IR-UTE images at TE = 0.02 ms. In the top right figure, the red ROI corresponds to the genu corpus callosum and the blue ROI corresponds to the ventral hippocampal commissure. **(B)** Average magnitude of IR-UTE images at TE = 0.02 ms for selected ROIs. Bars represent 95% confidence intervals. (a.u.), arbitrary units.

The IR-UTE signal visually decreased in the cuprizone-treated mice in several white matter tracts that are known to be sensitive to cuprizone-induced demyelination (Figure 2). Axial images through the ventral hippocampal commissure and splenium of the corpus callosum demonstrate the greatest decrease in signal intensity in the corpus callosum and to a lesser extent in the ventral hippocampal commissure and white matter of the basal ganglia. In contrast, there was no significant signal change in the internal capsule, which is known to be resistant to cuprizone (Yang et al., 2009). These findings are confirmed to be statistically significant with quantitative analysis using ROIs (Figure 3).

**Figure 4:**
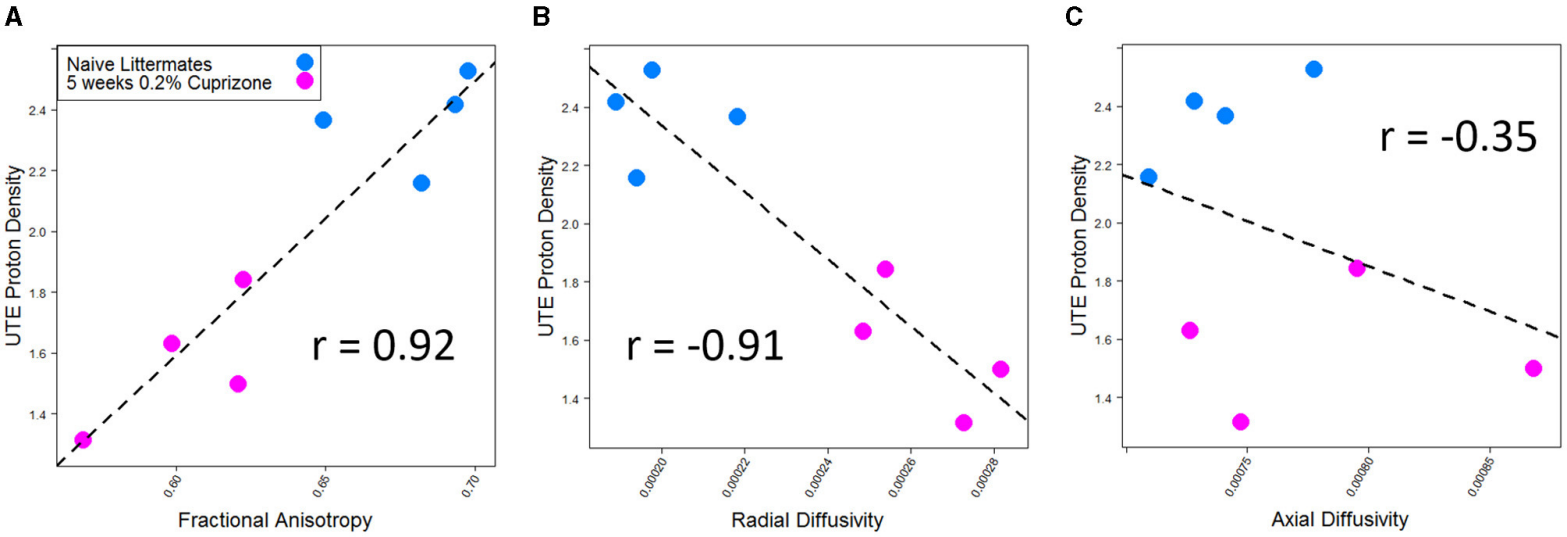
IR-UTE signal correlates with **(A)** fractional anisotropy and **(B)** radial diffusivity, but not **(C)** axial diffusivity in cuprizone-treated mice. Both fractional anisotropy and radial diffusivity but not axial diffusivity were different between cuprizone-treated and untreated mice with *p* < 0.05.

DTI imaging also was able to detect a difference between the control and cuprizone-treated mice. Demyelination is thought to be associated with a decrease in total fractional anisotropy, specifically with an increase in radial diffusivity as the loss of myelin bilayers allows water molecules to diffuse in a radial direction from the axon tracts (Xie et al., 2010). In contrast, axial diffusivity is more sensitive to axonal damage, which transiently changes during early treatment with cuprizone (Sun et al., 2006). As expected and in line with prior studies of DTI imaging of cuprizone-treated mice, a decrease in fractional anisotropy and an increase in radial diffusivity were seen in the splenium of the corpus callosum, whereas there was no statistically significant change in axial diffusivity. The IR-UTE signal correlated with both fractional anisotropy and radial diffusivity, but only had a weak correlation with axial diffusivity that was not statistically significant (Figure 4).

**Figure 5:**
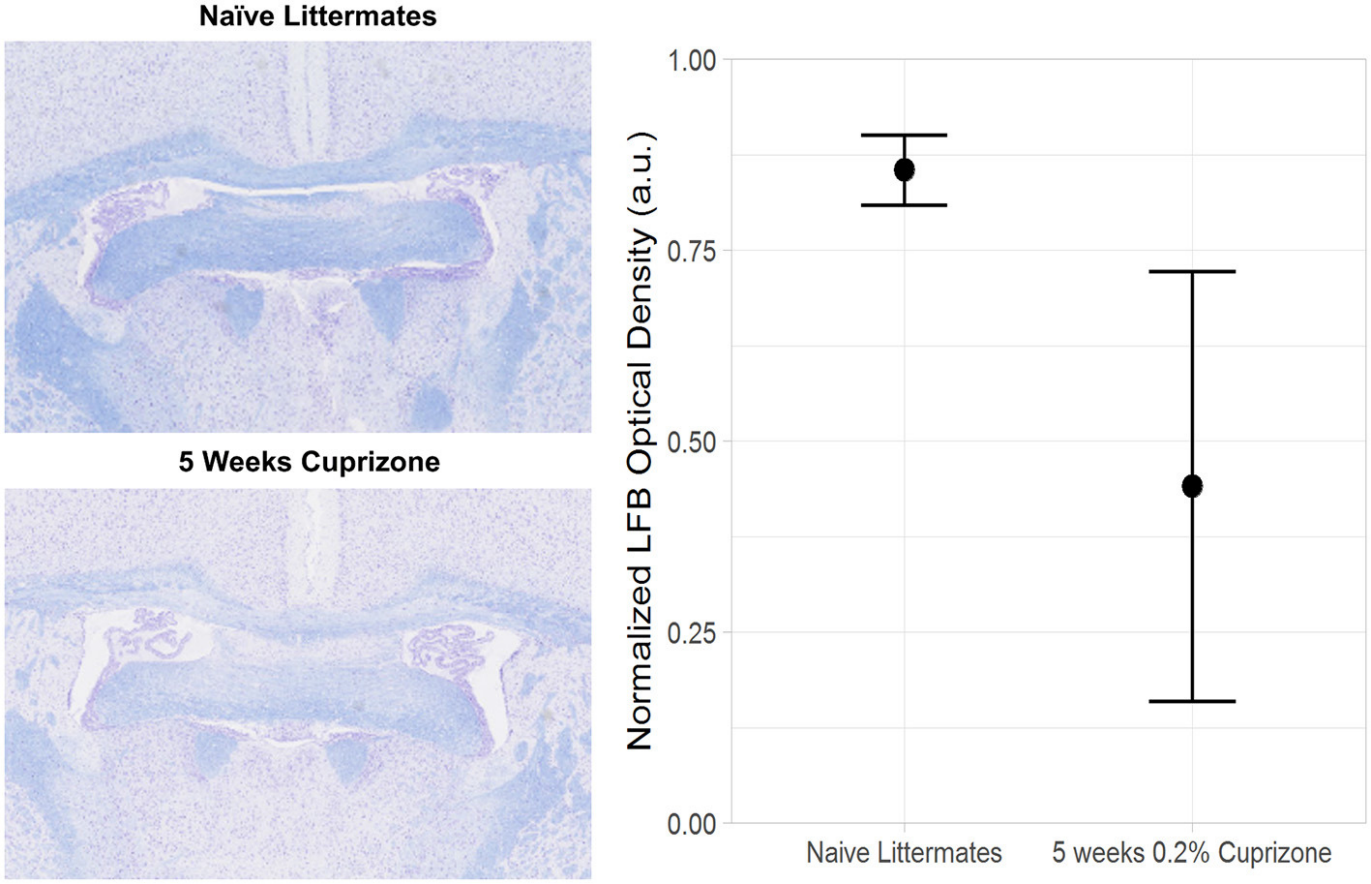
Decreased Luxol Fast Blue (LFB) staining in cuprizone treated mice compared with control mice. The plane of sectioning is analogous to Figure 2A, showing decreased myelin in both the genu of the corpus callosum and the ventral hippocampal commissure.

**Figure 6:**
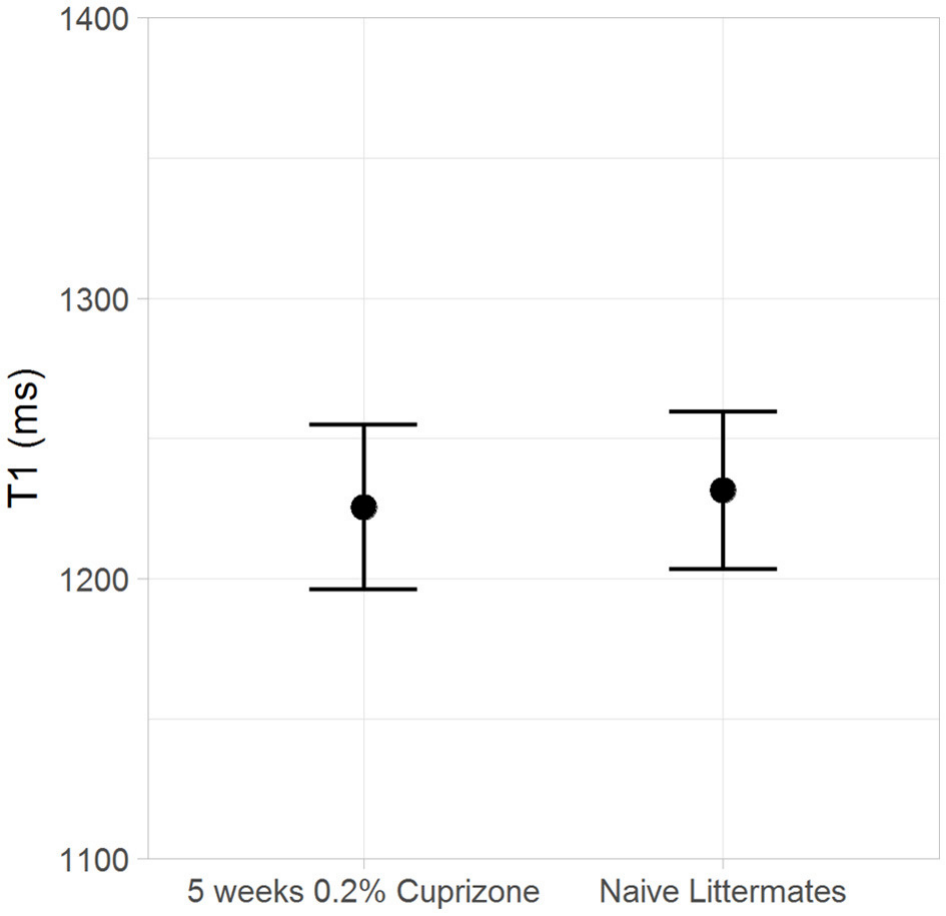
The T1 of myelin in the ventral hippocampal commissure is unchanged by cuprizone treatment.

Both the IR-UTE and DTI imaging results are corroborated by semi-quantitative histochemical analysis of myelin using LFB staining (Figure 5). All of the cuprizone-treated mice had decreased myelin staining in the genu of the corpus callosum compared to the control mice; however complete demyelination was not achieved in this study. Additionally, T_1_ mapping demonstrates that cuprizone treatment did not significantly change the T_1_ of the ventral hippocampal commissure (Figure 6). Therefore, the TI_null_ of the long ${\text{T}}_{2}^{*}$ components does not significantly change in this model. Otherwise, it could have resulted in artifactual signal intensity changes.

## 4 Discussion

This study demonstrated that the 3D IR-UTE sequence is able to detect demyelination with decreased myelin signal in an animal model of acute demyelination. The ability of the IR-UTE sequence to detect the ultrashort T_2_ signal and suppress the long T_2_ signal was demonstrated in mouse brains, similar to prior work using *in vitro* phantoms and human volunteers (Du et al., 2014a; Sheth et al., 2017; Fan et al., 2018). The mice treated with cuprizone demonstrated loss of IR-UTE signal in white matter tracts known to be sensitive to cuprizone but no changes in white matter tracts that are known to be resistant, whereas the IR-UTE signal in white matter tracts of control mice was unchanged. The IR-UTE signal was also correlated with findings using DTI MRI. The 3D IR-UTE measurement of demyelination was further confirmed by LFB staining. These findings demonstrate that the IR-UTE signal was sensitive to myelin loss in the cuprizone mouse model.

Many of the existing methods for myelin imaging and quantification detect myelin indirectly through its interactions with myelin-associated water, and have been shown to detect demyelination in the cuprizone model and other animal models. These include magnetic transference (MT)-based imaging methods (Zaaraoui et al., 2008; Varma et al., 2015; Khodanovich et al., 2017), T_2_ relaxometry/myelin water fraction imaging (Thiessen et al., 2013; Wood et al., 2016), diffusion based imaging methods (Song et al., 2005; Sun et al., 2006; Wang et al., 2011), quantitative susceptibility mapping (Wang et al., 2019), and others. However, these methods may also be sensitive to other pathologic changes including microgliosis, edema, and mild axonal injury that accompany demyelination in this model (Wood et al., 2016). Directly imaging myelin protons using IR-UTE would be expected to improve specificity for myelin loss in the setting of heterogeneous pathological changes, or may complement these other methods. The indirect measures of myelin are sensitive to B_1_ and B_0_ inhomogeneities and may be complicated by edema and iron deposition. Unlike IR-UTE, conventional MRI techniques also cannot measure myelin relaxation times (e.g., T1 and T2^*^ relaxation times), which may allow for a direct assessment of myelin quality.

Recently, another UTE-based method was found to correlate with histological markers of myelin in the cuprizone model better than the myelin water fraction and RD and similar to the MT-based macromolecular fraction (Soustelle et al., 2019). This Diff-UTE sequence uses diffusion gradients for suppression of long T_2_ water signals, which allows relative preservation of the ultrashort T_2_ signal at a short TR. In contrast, the IR-UTE sequence uses IR preparation for long T_2_ signal suppression, allowing for recovery of the ultrashort T_2_ signal using a longer TR and multispoke acquisition per IR preparation (Carl et al., 2016; Ma et al., 2020b). The IR-UTE sequence is more robust to B_1_ inhomogeneity and does not require assumptions about the T_1_ and T_2_ of myelin for gradient tuning, which is required in Diff-UTE imaging of myelin for appropriate signal nulling of diffusive long-T2 components of the white matter (Soustelle et al., 2019); however, inversion time needs to be carefully determined for IR-UTE imaging. We have recently designed a Double-Echo Sliding Inversion Recovery Ultrashort Echo Time (DESIRE-UTE) method which allows image reconstruction at a wide range of inversion times so that the optimal inversion time does not need to be chosen prospectively (Ma et al., 2020c). Another approach is a short-TR adiabatic inversion-recovery UTE (STAIR-UTE) method which allows robust long T_2_ signal suppression with optimized short TR/TI pairs (Ma et al., 2020a). In addition, the Diff-UTE study was performed on mouse brains that had been fixed, which may alter the MR properties of myelin. The specificity of the IR-UTE and Diff-UTE sequences have not yet been tested during remyelination or in other models of demyelination.

There are several limitations of this study. First, the sample size of both the treated and control mice was small, owing to the fact that this study was designed as a proof-of-concept for future studies. Additionally, complete demyelination was not achieved, which limited the ability to examine other contributors to the ultrashort T_2_ IR-UTE signal such as inflammation and gliosis. A possible reason for myelin not being as low as expected could be animals not eating enough, or variable amounts, of the cuprizone-containing diet. Future studies will be needed to test the specificity of the IR-UTE signal for myelin in the setting of both demyelination and remyelination using other models of demyelination, and to investigate the advantages and disadvantages over conventional MRI techniques for myelin quantification.

## 5 Conclusion

In conclusion, the 3D IR-UTE method was able to robustly detect the ultrashort T_2_ components in major white matter tracts of the mouse brain with decreased IR-UTE signal of myelin during cuprizone-induced demyelination as confirmed by LFB staining. Therefore, 3D IR-UTE is a promising non-invasive method for measuring brain myelin content in mouse models of demyelination.

## Data availability statement

The raw data supporting the conclusions of this article will be made available by the authors, without undue reservation.

## Ethics statement

The animal study was approved by IACUC at UCSD and at Novartis Institutes for BioMedical Research. The study was conducted in accordance with the local legislation and institutional requirements.

## Author contributions

ACS: Conceptualization, Data curation, Formal analysis, Investigation, Methodology, Software, Writing—original draft, Writing—review & editing. YM: Conceptualization, Data curation, Formal analysis, Investigation, Methodology, Software, Writing—original draft, Writing—review & editing. SrihS: Resources, Supervision, Writing—review & editing. SrinS: Resources, Supervision, Writing—review & editing. RB: Data curation, Methodology, Resources, Software, Writing—review & editing. EC: Conceptualization, Funding acquisition, Methodology, Resources, Supervision, Writing—review & editing. LD: Investigation, Methodology, Resources, Writing—review & editing. AMS: Investigation, Methodology, Resources, Writing—review & editing. JD: Conceptualization, Funding acquisition, Methodology, Project administration, Resources, Supervision, Writing—original draft, Writing—review & editing.
